# Pollen Allergies in Humans and their Dogs, Cats and Horses: Differences and Similarities

**DOI:** 10.1186/s13601-015-0059-6

**Published:** 2015-04-07

**Authors:** Erika Jensen-Jarolim, Lukas Einhorn, Ina Herrmann, Johann G Thalhammer, Lucia Panakova

**Affiliations:** Comparative Medicine, Messerli Research Institute of the University of Veterinary Medicine Vienna, Medical University Vienna and University Vienna, c/o Institute of Pathophysiology and Allergy Research, Währinger G. 18-20, Vienna, 1090 Austria; Dermatology Unit, Clinics of Small Animals and Horses, University of Veterinary Medicine, Vienna, Austria

**Keywords:** Pollen allergy, Human, Canine atopic dermatitis (CAD), Dog, Feline, Cat, Equine, Horse, Allergy diagnosis, Allergen immunotherapy

## Abstract

Both humans and their most important domestic animals harbor IgE and a similar IgE receptor repertoire and expression pattern. The same cell types are also involved in the triggering or regulation of allergies, such as mast cells, eosinophils or T-regulatory cells. Translational clinical studies in domestic animals could therefore help cure animal allergies and at the same time gather knowledge relevant to human patients. Dogs, cats and horses may spontaneously and to different extents develop immediate type symptoms to pollen allergens. The skin, nasal and bronchial reactions, as well as chronic skin lesions due to pollen are in principle comparable to human patients. Pollen of various species most often causes allergic rhinitis in human patients, whereas in dogs it elicits predominantly eczematous lesions (canine atopic dermatitis), in horses recurrent airway obstruction or hives as well as pruritic dermatitis, and in cats bronchial asthma and so-called cutaneous reactive patterns (eosinophilic granuloma complex, head and neck pruritus, symmetric self-induced alopecia). In human allergy-specific IgE detection, skin tests or other allergen provocation tests should be completed. In contrast, in animals IgE and dermal tests are regarded as equally important and may even replace each other. However, for practical and economic reasons intradermal tests are most commonly performed in a specialized practice. As in humans, in dogs, cats and horses allergen immunotherapy leads to significant improvement of the clinical symptoms. The collected evidence suggests that canines, felines and equines, with their spontaneous allergies, are attractive model patients for translational studies.

## Introduction

### Human pollen allergy

**Figure 1:**
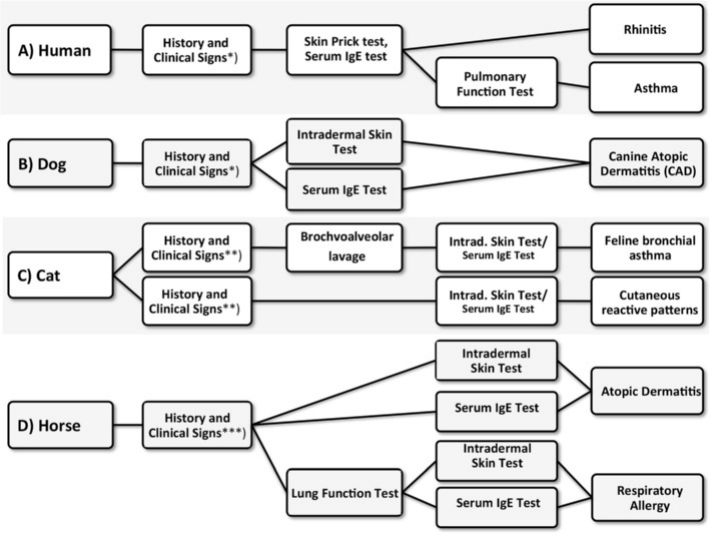
**Species-specific diagnostic methods to confirm pollen allergy.** Generally in allergic diseases of humans, dogs, cats and horses a careful anamnesis in context with the clinical picture is uttermost important. *) To consider differential diagnoses belongs to the primary work up of allergies; **) In pruritic cats consideration of metabolic, neoplastic, infectious and organic disorders is necessary because cutaneous reactive patterns are not pathognomic for allergy; ***) In horses it is especially important to rule out differential diagnoses like other skin diseases e.g. Erythema multiforme as an alternative cause for urticaria.

It is of great interest to compare the sensitization to pollen allergens and subsequent clinical manifestations between human patients and their domestic animals, such as dogs, cats and horses [1]. Generally, the sensitization to pollen allergens is high in Europe and ranges up to almost 70% in allergic human subjects [2]. For standard diagnosis the skin prick test (SPT) is an important cornerstone. In SPT an allergen is brought into the skin only epicutaneously, and a wheal and flare diameter above the positive control, or at 3 mm diameter, are counted as positive. The atopy patch test is a possible alternative [3]. Depending on the geographical exposure, accross Europe a panel of 18 allergens is needed for diagnosis, and there are efforts to standardize the panel of allergens used for diagnosis [4]. The diagnosis in human allergic patients is therefore based on history, skin test and determination of allergen-specific IgE, completed by nasal conjunctival, or pulmonary function tests (Figure 1). Whereas the overwhelming number of tests are still performed with allergen extracts, component-resolved allergy diagnosis is becoming increasingly routine for allergic human (but not animal) patients through single molecule CAP testing or the use of the ImmunoCAP ISAC112 microarray [5], where 28 of the 112 spotted molecules represent pollen allergen molecules.

In human patients rhinitis is the most prominent manifestation of pollen allergy, often in connection to conjunctivitis. Pollen allergies are associated with an oral allergy syndrome in 23% of cases [6]. More importantly, according to the “united airways” principle there is a significant correlation of allergic rhinitis with asthma, prompting the ARIA (Allergic Rhinitis and its Impact on Asthma) –initiative by the World Allergy Organization [7]. In human allergies pollen, in addition to house dust mites, animal dander and mold, play a dominant role. Pollen allergy has a more significant effect on the quality of life in patients than allergy to house dust mites, even up to several years after exposure [8], becoming worse when rhinitis is combined with asthma [9]. Pollen allergy can be effectively treated by allergen immunotherapy, thereby enhancing the quality of life [10].

## Review

### Pollen allergies in dog, cats and horses - seasonality

Generally, clinical signs of pollen allergy in these species tend to worsen seasonally if combined with other allergies, or in the case of pollen-only allergies, are strictly seasonal. Depending on the severity of clinical signs, a complete diagnostic work-up might be performed within the first season. In patients with a suggestive history and clinical signs of pollen allergy (see below for species-specific differences), the diagnostic work-up consists of ruling out any other differential diagnoses (e.g. parasites, flea allergy, food allergy and other species dependent disorders- e.g. Erythema multiforme in horses with signs of urticaria, or painful disorders in cats with self induced alopecia of the abdominal skin) (Figure 1). After this work-up, allergy testing using intradermal tests or serology (see below) is performed. In patients with known seasonal allergic disease in e.g. second season or even later, allergy testing is usually performed without ruling out other less probable differentials, such as food allergy.

### Pollen allergies in the dog *(Canis familiaris)*

**Figure 2:**
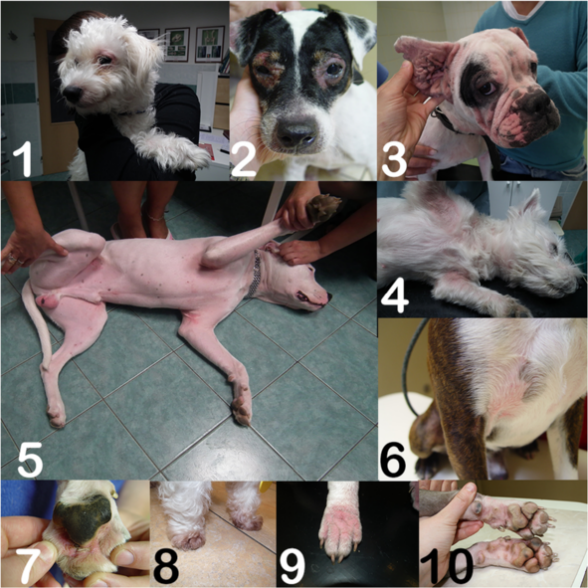
**Typical clinical pictures to illustrate canine atopic dermatitis.** In 1: Maltese; 2: periocular dermatitis in Jack Russel terrier; 3: English bulldoge; 4: West Highland White terrier (WHW); 5: Doggo Argentino; 6: Boston terrier; Bottom panel: atopic pododermatitis in 7: French bulldoge; 8: WHW; 9: English bulldoge; 10: Pitbull.

**Figure 3:**
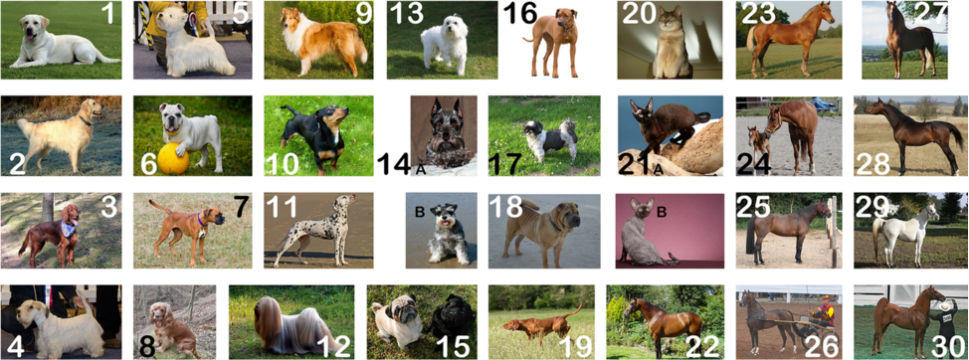
**Illustration of the most important breeds of dogs, cats and horses that were described to have a higher risk for atopic/allergic diseases (accessory to** **Table** 1 **). 1. Labrador Retriever:** ©Michael Bernkopf. **2. Golden Retriever:** from Pixabay. **3. Setter:** from Pixabay. **4. Sealyham Terrier:** ©Michael Bernkopf. **5. West Highland White Terrier (WHW):** © Michael Bernkopf. **6. English Bulldog:** from Pixabay. **7. Boxer:** from Pixabay. **8. Cocker Spaniel:** from Pixabay. **9. Collie:** from Pixabay. **10. Foxhound:** from Pixabay. **11. Dalmatian:** from Pixabay. **12. Lhasa apso:** from Lhasaapso (Own work) [CC BY-SA 3.0 (http://creativecommons.org/licenses/by-sa/3.0)], via Wikimedia Commons. http://commons.wikimedia.org/wiki/File%3ALhasaapso.jpg. **13. Maltese:** from Pixabay. **14 A Miniatur Schnauzer:** from Pixabay. **B Schnauzer** from Pixabay. **15. Pug:** from Pixabay. **16. Rhodesian Ridgeback:** from Pixabay. **17. Shi-TZU:** from Pixabay. **18. Shar peis:** from Flickr: M.Peinado Commons: M.Peinado (originally posted to Flickr as Gorda - 100) [CC BY 2.0 (http://creativecommons.org/licenses/by/2.0)], via Wikimedia Commons. http://commons.wikimedia.org/wiki/File%3AGorda_-_100.jpg. **19. Vizsla:** from Noveczki Katalin (own work) [GFDL (http://www.gnu.org/copyleft/fdl.html) or CC BY-SA 3.0 (http://creativecommons.org/licenses/by-sa/3.0)], via Wikimedia Commons. http://commons.wikimedia.org/wiki/File%3AVizsla_r%C3%A1h%C3%BAz_a_vadra.jpg. **20. Abyssinian cat:** from Pixabay. **21. A Devon cat:** Fergie, kindy provided by Sybille Greff. **B Devon cat:** Lakritze, kindy provided by Sybille Greff. **22. Dutch warmblood:** “Ubility” by Remy Overkempe - originally posted to Flickr as Ubility. Licensed under CC BY-SA 2.0 via Wikimedia Commons - http://commons.wikimedia.org/wiki/File:Ubility.jpg#mediaviewer/File:Ubility.jpg. **23. Silver Morgan:** By Laura Behning. [CC BY 2.0 (http://creativecommons.org/licenses/by/2.0)], via Wikimedia Commons. http://commons.wikimedia.org/wiki/File%3ASilverMorgan.jpg. **24. Swedish Warmblood:** By Jenny Dybedahl (Own work) [GFDL (http://www.gnu.org/copyleft/fdl.html) or CC BY-SA 3.0 (http://creativecommons.org/licenses/by-sa/3.0)], via Wikimedia Commons. http://commons.wikimedia.org/wiki/File%3AFoal-three-hours-old.jpeg. **25. Oldenburg horse:** By Martin Bahmann (Own work) [GFDL (http://www.gnu.org/copyleft/fdl.html) or CC-BY-SA-3.0 (http://creativecommons.org/licenses/by-sa/3.0/)], via Wikimedia Commons. http://commons.wikimedia.org/wiki/File%3ABess2.jpg. **26. Hackney Pony:** by Heather Moreton from Louisville, KY, USA (Hackney Pony Uploaded by Princess Mérida) [CC BY 2.0 (http://creativecommons.org/licenses/by/2.0)], via Wikimedia Commons. http://commons.wikimedia.org/wiki/File%3AHackney_Pony_(7714709846).jpg. **27. Paso Fino:** by Arsdelicata (Own work) [CC BY-SA 3.0 (http://creativecommons.org/licenses/by-sa/3.0) or GFDL (http://www.gnu.org/copyleft/fdl.html)], via Wikimedia Commons. http://commons.wikimedia.org/wiki/File%3APuerto_rican-Paso-Fino-Horse-chestnut.jpg. **28. Polish Arabian:** by Alina-Arabians (own work) [CC BY-SA 3.0 (http://creativecommons.org/licenses/by-sa/3.0) oder GFDL (http://www.gnu.org/copyleft/fdl.html)], via Wikimedia Commons. http://commons.wikimedia.org/wiki/File%3APoster_(Ekstern_x_Parella_-_Arbil).jpg **29. Arab, Mare:** by Pixabay. **30. Saddlebred American:** by Heather Moreton from Louisville, KY, USA [CC BY 2.0 (http://creativecommons.org/licenses/by/2.0)], via Wikimedia Commons http://commons.wikimedia.org/wiki/File%3AFive_Gaited_American_Saddlebred_(3007248363).jpg.

Pollen hypersensitivity is associated with Canine Atopic Dermatitis (CAD), a disease associated with high specific IgE against environmental allergens [11]. Generally, pollen sensitization is believed to be of minor impact in allergic dogs even though classical studies indicated similar nasal congestion symptoms in humans and dogs, for instance to ragweed pollen exposure [12]. The clinical picture differs from that of human rhinitis patients suffering from pollen allergy. In studies on nasal discharges in dogs, allergic rhinitis due to pollen allergens was excluded as a cause [13], rather nasal encounter with molds may play a role [14,15]. Thus according to veterinary practice, the canine allergic patient most often presents with pruritic allergic dermatitis (Figure 2). Here, differential diagnoses to e.g. food-induced allergic dermatitis have to be ruled out first. Either (for practical reasons) intradermal tests or determination of specific serum IgE lead then to the diagnosis of canine atopic dermatitis (Figure 1). Whenever pollen sensitization is diagnosed it may be associated with conjunctivitis in 21% of the dogs [16] and with rhinitis, but it is not associated with asthma [16,17]. The antigen-driven acute and chronic skin inflammation is usually termed CAD by the veterinary dermatologist, is seen independent of a true atopic background in the human sense, but associated with specific IgE [18]. The nomenclature of canine allergic diseases reflects that the skin is the most prominently affected organ: CAD, food induced allergic dermatitis (FIAD), ALD atopic-like dermatitis (ALD), or FAD (Flea Allergy Dermatitis); (asthma and anaphylaxis in dogs are only seen in experimental models, in rare cases anaphylaxis may be drug- or insect venom-induced). Like in humans sensitizations may be associated with an atopic predisposition, which in dogs strongly varies depending on the breed. In a US study 9% of 30.000 investigated dogs showed signs of CAD, among them a series of breeds being at higher risk [19] (Table 1, Figure 3), such as the Labrador [20], Maltese or Shih-Tzu in a Korean study - where in fact sensitizations were mostly found to indoor but not pollen allergens [21]. In a Swiss study, WHWT (White Wine terrier), boxer, French bulldog, Vizsla, bullterrier and Rhodesian ridgeback were at higher risk; additionally, pugs and Dalmatians were over-represented although without significance [22]. Atopic predisposition may also enable early sensitization to outdoor allergens, as was shown in classical [23] and novel dog models where ragweed exposure lead to an asthmatic phenotype [24].

**Table 1:** **Like atopic humans some domestic animals may have a higher genetic risk to develop allergies**

**Dogs** **[** 19 **-** 21 **]**	**Cats** **[** 41 **]**	**Horses** **[** 60 **]**
Retrievers (Labrador, Golden)	Abyssinian cat	Dutch Warmblood
Setters (English, Irish)	Devon rex cat	Morgan
Terriers (Boston, Cairn, Fox, Sealyham, Scottish, West Highland White, Wheaton)		Swedish Warmblood
Bulldog (French)		Oldenburg
Boxer		Hackney horse
Cocker Spaniel		Paso fino
Collie		Polish Arabian
Foxhound		Arab/Saddlebred cross
Dalmatian		
Lhasa Apso		
Maltese		
Miniature Schnauzer Pug		
Rhodesian ridgeback		
Shih-Tzu		
Shar Peis		
Vizsla		

Most important atopic breeds are illustrated in Figure 3.

Pollen allergen sensitization in dogs is evaluated by intradermal skin testing (IDT), injecting the allergen extracts into the shaved skin of the lateral thorax or abdomen [25]. Usually, the animals have therefore to be sedated or anesthetized. Reddishness, wheal and flare reactions of half of the size of the positive control (histamine) are interpreted as specific reaction. Generally, dogs react to pollen from grasses, trees and weeds (Table 2). The results of our literature research show a high variability in prevalence, probably caused by different geographic regions and lifestyle; insufficient data were found for cats and horses.

**Table 2:** Overview of types of pollen causing atopic/allergic diseases in domestic animals and respective references *)

**Species**	**Dog**	**Cat**	**Horse**
**Grasspollen**			
Orchard	82% [74]	8,3% [77]	
	50% [75]		
	3-8% [76]		
Timothy	76% [74]		
	15-16% [76]		
**Tree pollen**			
Birch	35% [75]	0% [77]	0% [78]
	14,6% [29]	4,1% [77]	
	5-10% [76]		
Ash	71% [74]		
	11,6% [29]		
	6% [76]		
Japanese Ceder	50% [31]		
Oak	78% [74]		
	12% [29]		
	14% [79]		
	7-16% [76]		
**Weed pollen**			
Ragweed	59% [75]	0% [77]	0% [78]
	13,6% [29]		
Mugwort	52% [75]	0% [77]	0% [78]
	11,9% [29]		
	9,6% [36]		
	6-10% [76]		
Red Clover	5-10% [76]		

*) The results show a high variability probably caused by different geographic regions and life style.

In contrast, a human allergist may get the impression that in veterinary practices IgE testing has a lower impact, due to a lack of standardized allergen extracts or reliable anti-dog IgE reagents. Interestingly, the human alpha chain of the high affinity receptor FcεRI is used in a commercial test for the detection of e.g. canine IgE [26]. This is possible due to a 54% amino acid identity and 68% similarity among the human and canine alpha chain and precise knowledge of the amino acids involved in the IgE binding [27]. To the best of our knowledge, the ISAC microarray testing has not been introduced into veterinary allergy diagnosis so far.

#### Specific sensitizations to pollen in canine versus human studies

In more than 1000 atopic dogs in Australia, a 10 to 25% sensitization to pollen of any kind (grass, tree, weed) was determined by intradermal tests [25]. A more recent cross-sectional study in 651 atopic dogs indicated that sensitization between tree, weed and grass pollen, but not to other allergen sources, were in 94% of cases statistically associated [28]. The authors pointed out that sensitization must clearly be distinguished from clinically relevant sensitization leading to symptoms.

No seasonal, sex or age dependent risk factors were observed in a recent comprehensive study of canine grass pollen sensitization in Western France [29]. Importantly, like in humans, a significant increase in the number of dogs sensitized to grass pollen was observed, namely 14,4% between 1999 and 2002, and 27,7% between 2007 and 2010. More than 80% of the 262 tests were positive for one allergen out of 20-38 extracts tested (among them 4 grass pollen, 8 weed pollen and 17 tree pollen) and 21% for at least one pollen allergen. The diagnosis of the genuine sensitizing allergen may be complicated by cross reactivities. Müller et al. proposed that, although positive reactions among botanically closely related plant allergens may be significantly more common than those among nonrelated allergens, cross reactivity in 30% of the tested dogs was not pronounced enough to warrant testing and desensitization using allergen mixes [25,30].

Exposure to pollen depends on the local plant species, thus geographical differences enable distinct sensitizations. Masuda et al tested 42 Japanese atopic dogs by IDT and determined specific IgE using 26 allergen extracts from 8 allergen sources [31]. Japanese cedar (*Cryptomeria japonica*) pollen, after house dust mites, was the second most important allergen in this area, with a sensitization prevalence of 50% and a positive IgE reactivity of 16,7% of tested pet dogs. The same sensitization pattern was seen in the Japanese human patients [32], where besides house dust mites, cedar pollen has become an important health challenge [33]. In a more recent approach, the sensitization to single allergen molecules from Japanese cedar pollen were evaluated more precisely in a component-resolved manner in 15 dogs. Besides IgE to Japanese cedar molecules Cry j 1 and Cry j 3, 76% of tested dogs showed IgE to Cry j 6, hence identifying a new major canine allergen [34]. Interestingly, in canine pollen allergy, an oral allergy syndrome to related foods can occur likely due to cross reactivity between cedar pollen and tomato [35], although this has only been reported sporadically. The results from a study in the Bangkok area used a mix of 24 different pollen types in 114 atopic dogs [36], but in this population pollen allergens seem to play a minor role as compared to dust mite, cockroach, ant and other insect allergens. This mirrors the situation in Thai children where the prevalence of grass pollen allergy was below 5% as compared to 50% house dust mite and 23% cockroach sensitization [37].

#### Clinical treatment: SIT

Interestingly, CAD is regularly and with significant success treated by the veterinary dermatologist through allergen immunotherapy [38]. In fact, the International Task Force on Canine Atopic Dermatitis recommends offering SIT to each canine patient sensitized to environmental allergens including pollen [39]. In human atopic eczema treatment SIT is a controversial topic, but here as well it may improve allergic symptoms even in settings of atopic predisposition [40]. Generally, the dose and frequency of injections in allergic dogs were, at least in 2001, less harmonized than in human allergology [41] and were more recently compared in [42].

### Pollen allergies in Cats *(Felis catus domestica)*

In a recent retrospective study of 45 Australian cats with atopic dermatitis, strong intradermal test reactions were most frequently seen to pollen allergens (61%) [43]. In fact, in pollen-allergic cats the skin is most often affected by pruritic allergic /atopic lesions, but cats may also present with allergic rhinitis, such as the Japanese cat which was diagnosed with cedar pollinosis [44]. Alternatively, cats with asthma may present with spastic coughing, where bronchoalveolar lavage as the next diagnostic step may reveal eosinophilic inflammation (Figure 1). Intradermal tests will lead then to the final diagnosis, whereas IgE determination has a lower impact.

The differential diagnosis in lower airway diseases associated with cough, respiratory distress, or both may be bronchitis or asthma, pneumonia, or neoplasms [45]. Feline asthma is characterized by eosinophilic inflammation and cats have been used as animal models for human asthma [46,47]. One study reported that domestic mixed breeds, Abyssinian and Devon rex cats are predisposed, compared to the population of the dermatology referral service [43]. The comprehensive central registry of all pet animals in Switzerland enabled the statistical evaluation in this study.

Clinical signs of cutaneous hypersensitivities in cats are not pathognomonic and include eosinophilic granuloma complex and self-induced hair-loss, and should be scored by an objective scale (SCORing Feline Allergic Dermatitis; SCORFAD) [48]. The AD in cats is not necessarily connected with the levels of IgE or the diagnosis of specific IgE antibodies in serum [49]. IgE to environmental allergens including pollen can be even found in pathogen-free housed cats [50,51]. When two groups of 10 cats each were intradermally tested, immediate reactivity was reported as IgE- as well as IgG-mediated reaction, but also explained by nonspecific mast cell degranulation [52]. The authors proposed the prior injection of a fluorescent agent in order to enhance the fidelity of interpretation, a procedure connected with substantially higher strain for the animals. Even in early studies specific IgG directed against ryegrass pollen, in addition to flea and house dust mite allergens, was reported and proposed as the second Th2 antibody class of relevance in feline allergy [53]. Rather than pollen, house dust mite allergens are found abundantly in the sleeping places of cats, but are not yet definitely proven to be causative allergens in feline AD or asthma [51].

Itchy inflammatory allergic skin diseases were successfully treated in 100 cats by daily oral immunosuppression with Cyclosporine over a six-week period [54]. Generally, allergen immunotherapy is described for cats, but is not yet standard care in veterinary dermatology. This is possibly due to the fact that the underlying IgE-based diagnosis for identification of the relevant allergen still lacks sufficient specificity [55]. Elimination diets, necessary for ruling out food hypersensitivities in cats and other species with non-seasonal cutaneous hypersensitivities, are usually performed prior to aeroallergen testing. These diets are more difficult to perform in cats than in dogs, so the owner often cannot follow through on one important step in diagnosing allergies. Another reason is that cats usually tolerate glucocorticoids much better than dogs. As mentioned above, Cyclosporine A is also a very useful drug in feline allergic dermatitis, since it is effective and achievable treatment option in these species [56,57].

A study using the human FcεRI alpha chain for detecting feline IgE [26], which has a 56% amino acid identity and 72% homology, resulted in the conclusion that IgE detection did not diagnose feline food or environmental allergies, but was effective in diagnosing insect hypersensitivities [49]. Pilot studies with rush allergen immunotherapy with good tolerability were described in 4 cats [58]. In 81 cats SIT significantly improved the dermatological conditions between 93.6% (linear granuloma) and 60% (self-induced hair loss), with an 86.1% improvement in asthma [59]. Studies on subcutaneous versus mucosal (nasal) allergen immunotherapy or novel adjuvants were done in feline allergic asthma models, including Bermuda grass pollen. The authors reported improvement of the asthma, increase of the IL-4:IFN-gamma ratio and decline of bronchial eosinophils, as well as good tolerability [46]. Reinero et al. reported that in cats allergen immunotherapy may be associated with induction of T-regulatory cells and IL-10, and also cross-protect to non-related allergens, such as Bermuda grass to house dust mite allergens [60].

### Do horses *(Equus caballus domesticus)* suffer from pollen allergies?

In fact, horses do develop respiratory and skin diseases (urticaria, atopic eczema) due to allergens. Equine IgE can be detected by *in vitro* diagnosis. In this case the human alpha chain can also be used due to its 64% amino acid identity and 76% homology to the equine counterpart as evaluated by our own BLAST search. Moreover, in horses allergen immunotherapy is regularly done [61]. Intradermal allergy tests or serum IgE tests may be chosen for further work-up and diagnosis for either skin or respiratory allergy (Figure 1).

Again, several breeds can be called atopic and therefore are more prone to allergies; in one study, Dutch warmbloods, Morgans, Swedish Warmbloods, Oldenburgs, Hackney horses, Paso finos, Polish Arabians and Arabian/Saddlebred cross were overrepresented [62] (Table 1, Figure 3). In equine allergic respiratory disease the symptoms may range from rhinitis and asthma to chronic dry cough and emphysema [63], indicating that numerous immediate-type, delayed-immune complex or cellular hyperreactivities can be causative in acute recurrent airway obstruction (RAO) or chronic obstructive pulmonary disease (COPD). For diagnosing RAO a lung function test is helpful. Positive correlations between symptom severity and exogenous factors such as climatic conditions, rainfall and seasonal pollen counts have been observed [64]. Although recurrent obstruction may be clearly associated with the pollen season, the terminology is ‘obstructive pulmonary disease’, not asthma [65].

Additionally, pruritic skin diseases as well as recurrent urticaria may be observed in atopic horses. Furthermore, atopic dermatitis in equines is becoming a more commonly recognized disease, especially due to newer diagnostic methods and treatment options. It is interpreted in horses as an inherited predisposition to form specific antibodies to environmental allergens such as pollens of grasses, weeds and trees, but also to mold and dust. Clinical signs are pruritus and secondary intense self-trauma, crusting, alopecia with chronic lesions including lichenification and hyperpigmentation. Affected areas are often the ears, face, ventrum and legs. The clinical signs are similar to those caused by insect hypersensitivity and it is extremely common to have both diseases in the same horse [66].

Diagnosis of hypersensitivity in horses can be made based on clinical symptoms, ruling out other differential diagnoses (e.g. parasites, food hypersensitivities and insect hypersensitivities especially in the dermatologic patients), and by serology with specific IgE diagnosis and intradermal testing (Figure 1). It is substantial to differentiate between asthma and recurrent airway obstruction (RAO), as for the latter the contribution of IgE or IgG is still a matter of debate. In a recent study “reaginic” antibodies of the IgE and IgG class were described as participating in the pathophysiology of asthma and RAO [66], but also delayed type reactions were reported [67]. Equine RAO studies mostly identified Aspergillus as a prominent allergen and include today component-resolved diagnosis [67,68]. It may be anticipated that a similar pathophysiological principle, IgE and IgG, will possibly account for pollen allergy. Later studies suggest that better diagnostic tools could improve the fidelity of equine allergy diagnosis. In an approach with 64 horses specific reactivity could be found to grass pollen allergens when antisera to peptides from equine IgE were used [69].

Heaves is a recurrent neutrophilic lung disease in horses with clinical similarity to asthma [70] but likely caused by molds from airborne dust and not by pollen [71]. Interestingly, an enhanced apoptosis rate in CD4 and CD8 T-cells are seen in horses affected by RAO [72].

SIT can be performed in horses. For instance, 54 horses suffering from atopic skin diseases including urticaria and pruritus were treated with SIT successfully [62]. Although 75% of horse owners decided to discontinue after a period of between 6 months and 8 years, approximately half of the discontinued horses profited from the therapy and stayed free from recurrence of clinical signs. The antigens used were extracts from Arizona cypress pollen, red cedar pollen, in addition to sheep epithelia, box elder, house dust mix, Dermatophagoides farinae, Dermatophagoides pteronyssinus, horsefly, flea, Culicoides, black ant, corn, grain mill dust and grain smut. Of the 27 horses that were reported to benefit from SIT, 13 horses had their SIT formulated based on the results of IDT, nine had their SIT based on a serum test, and five had both an IDT and a serum test. A chi-square analysis used to compare the success proportions of SIT between skin tests, serum tests and both showed no statistical difference between the three groups.

## Conclusions

Pollen allergens in human allergic patients are mainly responsible for rhinitis and asthma, whereas in canines they predominantly cause canine atopic dermatitis, in cats rhinitis, asthma and dermatitis, and in horses recurrent urticaria, pruritic dermatitis and recurrent airway obstruction (in the latter species not called asthma). Generally, allergenic pollens that cause human disease are relevant for our domestic animals, at least for dogs (Table 2). The human and veterinary diagnosis differs slightly depending on the species, but clinical pictures are similar (Figures 1 and 2). Several breeds are at a significantly higher risk of developing allergic diseases (Table 1, Figure 3). IgE testing with extracts or molecules represents an indispensable cornerstone in human allergy diagnosis, but has not yet reached the same high fidelity in IgE testing of allergic dogs, cats and horses. This may be due to distinct pathomechanisms or a lack of optimized diagnostic tools. Therefore, in animals intradermal tests rather than epidermal prick tests are important tools for allergy diagnosis. In animals, but not in human allergic patients, the IgE test and intradermal test are regarded equal and may replace each other [73]. Allergen immunotherapy is a reliable instrument to reduce clinical symptoms both in humans and their allergic domestic animals.

## Abbreviations

ALD: Atopic-like dermatitis
ARIA: Allergic Rhinitis and its Impact on Asthma
CAD: Canine atopic dermatitis
COPD: Chronic obstructive pulmonary disease
FAD: Flea allergy dermatitis
FIAD: Food induced allergic dermatitis
IDT: Intradermal skin test
RAO: Recurrent airway obstruction
SCORFAD: SCORing feline allergic dermatitis
SIT: (allergen-) specific immunotherapy
SPT: Skin prick test
